# *Strengthening Medicare*: Will increasing the bulk-billing rate and supply of general practitioners increase access to Medicare-funded general practitioner services and does rurality matter?

**DOI:** 10.1186/1743-8462-2-18

**Published:** 2005-08-20

**Authors:** Susan E Day, Katrina Alford, David Dunt, Stuart Peacock, Lyle Gurrin, Don Voaklander

**Affiliations:** 1 Centre for Health and Society, School of Population Health, University of Melbourne, Australia; 2 Program Evaluation Unit, School of Population Health, University of Melbourne, Australia; 3 Cancer Control Research Program, British Columbia Cancer Research Centre, Canada; 4 Centre for Molecular, Environmental, Genetic and Analytic Epidemiology, School of Population Health, University of Melbourne, Australia; 5 Department of Public Health Sciences, University of Alberta, Canada

## Abstract

**Background:**

Recent increases in the bulk-billing rate have been taken as an indication that the Federal government's *Strengthening Medicare *initiative, and particularly the bulk-billing incentives, are 'working'. Given the enduring geographic differences in the supply of general practitioners (GPs) it is timely to reconsider the impact that this increase in the provision of 'free care' will have on access to Medicare-funded GP services in rural and urban areas of Australia. Utilisation has been modelled as two different stochastic processes: the decision to consult and the frequency of consultation.

**Results:**

In the decision to consult model the supply of FFS GPs is a more important predictor of utilisation than the bulk-billing rate. Paradoxically the modelling predicts that ceteris paribus increases in either GP supply or the bulk-billing rate appear to have perverse effects in some areas by decreasing utilisation. In the frequency of consultation model, GP density is not a predictor and increasing the bulk-billing rate will unambiguously increase the frequency of consultation across all areas. In both models, the positive impacts associated with changes in supply and cost are constrained outside the inner metropolitan area by reduced geographic accessibility to Medicare-funded GP services. The modelling also shows that people are more likely to consult a GP in areas of high socioeconomic disadvantage, although socioeconomic status is not a predictor of frequency of consultation.

**Conclusion:**

Bulk-billing rates and the supply of FFS GPs are important features of the Australian health care system that are, potentially, amenable to policy manipulation. The implications of this research are that government policies designed to achieve similarity in these characteristics across geographic areas will not result in equity of access because they fail to address problems caused by geographic inaccessibility in rural and remote areas. Attempting to increase bulk-billing rates in some of these areas may, in fact, reduce access to FFS GP services.

## Background

In April 2003 the Federal government announced a policy initiative called *A Fairer Medicare*. This initiative was heavily criticised and enabling legislation failed to pass the Senate. A revised version of the policy, called *Medicare Plus*, was announced in November 2003. Further revisions ensued before *Medicare Plus *was passed by the Federal parliament in March 2004 and its name was subsequently changed to *Strengthening Medicare *[1]. *Strengthening Medicare *aimed to improve access (including affordability) to out-of-hospital medical services and contained twenty-seven separate measures including incentive payments for general practitioners (GPs) to encourage bulk-billing of concession card holders and children under sixteen years (particularly in regional, rural and remote areas) and a series of measures to attract GPs to work in areas of undersupply such as outer metropolitan, regional and remote areas [1,2].

Although *Strengthening Medicare *and its precursors were not specifically aimed at increasing the bulk-billing rate, there was considerable debate about the impact that the initiatives would have on the bulk-billing rate and access to GP services (see for example, the first and second reports of the Senate Select Committee on Medicare) [3,4]. Also, one of the effectiveness indicators developed by the Department of Health and Ageing to measure progress towards Departmental outcomes in relation to access to Medicare is 'the percentage of Medicare services that are direct billed with no gap charged' [2]. Recent increases in the overall bulk-billing rate for GP services have been seen as an indication that the *Strengthening Medicare *package, and bulk-billing incentives in particular, are 'working' [1,5]. Given the recent increases in the level of bulk-billing (i.e. 'free care') provided by fee-for-service (FFS) GPs and enduring geographic differences in the supply of GPs, it is pertinent to question what impact increases in the bulk-billing rate will have on access to Medicare-funded GP services in different parts of Australia.

The most widely accepted study examining the relationship between price and utilisation of doctors' services is the Rand Health Insurance Experiment (HIE) in the United States. HIE results show that utilisation of outpatient services decreases when patients are required to pay a co-payment and that there are no differences in the nature of the response across geographic areas [6]. Richardson applied the HIE results to Australian Medicare data and concludes that 78% of the change in GP service utilisation in the 1976–1986 period and 94% of the change in the 1984/85–1989/90 period were not related to decreases in consumer co-payments – other factors were at work [7].

A review of Australian cross sectional studies examining factors associated with GP consultation rates indicates that higher frequencies are associated with lower cost, [8-10] patients having a health care card, [11] patients being in poorer health, [9,11] and with areas of higher proportions older age residents [10]. Lower frequencies are to be found in areas with higher levels of education [8] and among female patients with higher levels of education, [9] in less geographically accessible (eg rural) areas [10,11] and among female patients with an internal Health Locus of Control [9]. One study with highly aggregated levels of data showed an increase in the frequency of consultation in low socioeconomic status areas [12]. Another indicated that the relationship was not so straightforward. Low socioeconomic status was associated with an increase in the consultation rate in highly accessible areas and low consultation rates in inaccessible areas, [13] suggesting supply side factors are also important.

Cross sectional results for the impact of GP supply generally indicate an increase in supply being associated with an increase in frequency of consultation [10,14,15]. One Australian study by Doessel [16] indicated there was no association between GP supply and population based frequency of utilisation rates. However, Richardson and Peacock have indicated that these results should be interpreted carefully [15].

Observed associations between increases in doctor supply and increases in frequency of utilisation have given rise to the notion of supplier induced demand (SID). SID suggests that doctors are imperfect agents and can induce demand for health care, which directly conflicts with the full information and consumer sovereignty assumptions of the orthodox model of demand and supply [17]. Volumes of empirical evidence addressing the possibility of SID have been presented from a range of different health systems, and have been reviewed elsewhere [18-20]. Many of the studies reviewed have used cross-sectional data sets to examine the effect of doctor supply on the frequency of utilization of health care services [14,21-24].

However, studies examining only the frequency of utilisation do not take into account the nature of the decision making process in the demand for non-emergency care which is thought to reflect sequential decisions involving both the individual and the GP [25]. Initially an individual decides whether to seek out health care or advice. This decision to consult is derived from the demand for health [26] and reflects an individual's beliefs about the severity of their condition, the availability of health care and their expectation of any potential benefits. Once the contact decision has been made and acted upon, the individual and the doctor decide on the type and amount of health services to be provided and the frequency of future consultations. Although the decision about the type, amount and frequency of future consultations is made primarily by the GP [6,27,28] the patient also has a role in this decision. The extent to which the patient participates in the decision depends on the nature of the doctor-patient agency relationship [29].

A small number of studies have attempted to examine the effect of the supply of doctors upon the patient-initiated and doctor-initiated components of utilisation. The results of these studies are inconclusive. Rossiter and Wilensky found that increases in doctor supply had no effect on patient-initiated visits but did have a small effect on doctor-initiated visits and took this to be evidence of SID [30,31]. On the other hand, Escarce found that increased availability increased initial contacts but had little effect on the intensity of subsequent visits, the doctor-initiated component [32]. There is only one Australian study examining the patient-initiated component of the utilisation process. This study found a higher proportion of the resident population consulting a GP in areas with a greater supply of GPs, areas which have higher proportions of younger and older age residents, and areas of lower cost (higher bulk-billing rates). Lower proportions were associated with rural areas [10].

One of the shortcomings of much of the Australian work on the relationship between cost, supply and utilisation of GP services is the inability of the studies to account for border-crossing by patients. There is ample evidence that patients do not necessarily visit the nearest GP when they decide to see a doctor [33,34]. The studies also indicate significant interactions between the variables associated with utilisation of GP services yet these interactions do not appear to have been systematically explored. And the studies are not always inclusive: some target specific demographic groups, others omit certain age groups or geographic areas from their analyses. Finally, despite their widespread acceptance, [35] the generalisability of the HIE results has been questioned [36,37] and their relevance to the Australian situation is not clear.

This research uses publicly accessible data to investigate the likely impact of an increase in the bulk-billing rates and GP supply on access to Medicare-funded GP services in rural and urban areas. The methodology is based on an analysis of aggregated data from various sources and has been developed to be inclusive of all geographic areas and age groups, to take into account border crossing by patients, to systematically explore the interactions among the variables and to accommodate the two-part decision-making process thought to underlie the utilisation of GP services.

## Methods

### Unit of analysis

Divisions of General Practice consist of 'geographically co-located group(s) of general practitioners who have formed an organization to work together ... to improve health outcomes at the local level' and provide GPs with a 'corporate identity' as well as a 'method of influencing the organization of health care delivery' [38]. Divisions can, therefore, be regarded as geographically defined service delivery systems and are appropriate aggregation units for modelling the relationship between price, supply and utilisation of health care services. One hundred and twenty-one Divisions are included in the analysis.

### Data

Two utilisation models have been developed. The first reflects the decision to consult an FFS GP and the second reflects the frequency of consultation once the decision to consult has been made. These contact and frequency decisions are modelled as two different stochastic processes [28]. Failure to do this can lead to inconsistent parameter estimates and misinterpretation of results [25].

#### Decision to Consult Model

A patient to population ratio (decision to consult index) was considered an appropriate outcome variable for the contact model and, to overcome the problems of border crossing, the index has been defined as the number of Whole Patient Equivalents (WPEs) per head of resident population. (See the notes to Table 1 for the definition of WPE.)

**Table 1:** Variables and sources of data for the regression models

**Variables**	**Data Sources**
**Dependent: decision to consult index**	
Whole Patient Equivalents (WPEs) per head of population*	WPEs: Table S2 in the Statistical Appendix to the report *The General Practice Workforce in Australia*† Population: HealthWIZ v6.2‡ based on 1996 census
**Dependent: frequency of consultation**	
Group A1 and A2 consults per Standardized Whole Patient Equivalent (SWPE)^¶^	Group A1 and A2 Consultations: available at accessed Sept 7 2003 SPWEs: Table S2 in the Statistical Appendix to the report *The General Practice Workforce in Australia*†
**Predictor variables**	
Geographic accessibility: Population weighted ARIA values for each Division	% Population in postcodes for each Division: available at accessed Jul 11 2003 Population in each postcode: HealthWIZ v6.2‡ based on 1996 census ARIA values for postal areas originally accessed at accessed 26 Feb 2002. This url is no longer available but information about the data can be found at .
Bulk billing rate for general practice consultations	HealthWIZ v6.2‡
Dr Density: number of GPs and Other primary medical care doctors per 1,000 head of population	Vocationally Registered GPs and Other primary care practitioners: Table S2 in the Statistical Appendix to the report *The General Practice Workforce in Australia*†, derived from the 1998 Medical labour force survey undertaken by the Australian Institute of Health and Welfare (AIHW), Canberra Population: HealthWIZ v6.2‡ based on 1996 census
Index of Disadvantage	Population at each level of disadvantage within a Division: HealthWIZ v6.2‡
% population born in NESB country; % female, av age	HealthWIZ v6.2‡ based on 1996 census

**Notes**: * WPEs are derived by the GP Branch of the Commonwealth Department of Health and Ageing as an indicator of patient load for each practice. If a patient consulted at only one general practice during a financial year, the patient is counted as one WPE for the practice. If a patients visits more than one general practice, the patient is counted as a fraction of a WPE based on the schedule fee value for each general practice consulted.

† Australian Medical Workforce Advisory Committee: **The General Practice Workforce in Australia**. AMWAC Report 2000.2.

‡ Commonwealth Department of Health and Aged Care, National Social Health Statistical Database, developed by Prometheus Information Pty Ltd, commonly known as HealthWIZ. See Other Products at

^¶ ^SWPEs are standardised WPEs. 'The standardisation process is based on National Medical and Department of Veterans' Affairs claims figures for each of 16 age/gender categories. The standardisation is achieved by allocating each patient to one of sixteen categories and multiplying the WPE value for each patient by the appropriate weight.

#### Frequency of Consultation Model

Group A1 (General Practitioner) and Group A2 (Other un-referred) professional attendances are the basic building blocks of the Medicare system. In the 1998–99 financial year these services accounted for 95% of Medicare all services and 92% of the Medicare dollar benefits paid (excluding oral maxillofacial services) [39]. Frequency of consultation has been defined as the number of Group A1 and Group A2 services per Standardized Whole Patient Equivalent (SWPE). (See the notes to Table 1 for the definition of SWPE.)

Based on the review of the literature, the aim of the research and an exploration of the available data, the predictor variables initially entered into both models were: a Divisional measure of geographic accessibility, the bulk-billing rate, GP density, level of socioeconomic disadvantage of the resident population, and the proportion of the resident population born in a non-English speaking background (NESB) country. The proportion of the resident population that is female, and the average age of the resident population were also entered into the decision to consult model but not in the frequency of consultation model as age and gender are controlled for in the outcome variable. Sociodemographic information is based on the 1996 census and all other data relates to the 1998–99 financial year. The relevant data sources for the variables are shown in Table 1.

Assuming that Australian Bureau of Statistics postal areas are an adequate approximation of postcode areas, a population weighted geographic accessibility value was calculated for each Division by multiplying the Accessibility/Remoteness Index of Australia (ARIA) [40] for each postal area by the proportion of the Divisional population living in the matching postcode and then summing the results across all Divisional postcodes. Values range from zero to twelve and low values indicate greater geographic accessibility. The Divisional index of disadvantage has been derived from the 1996 Socio-Economic Indexes for Areas (SEIFA) Index of Disadvantage [41] statistics for each Division. Values of this index range from one to eleven and the original variable has been recoded so that higher values indicate higher levels of socioeconomic disadvantage. GP density is the number of vocationally registered plus other GPs per 1,000 head of resident population. Summary statistics for the variables used in the modelling are shown in Table 2.

**Table 2:** Summary of the dependent and predictor variables

		Highly Accessible* n = 64	Accessible* n = 37	Moderately Accessible* n = 7	Remote* n = 6	Very Remote* n = 5
**Dependent Variables**						
WPEs/head of pop^n^	Mean	0.95	0.83	0.76	0.70	0.54
	SD	0.09	0.06	0.10	0.05	0.12
Consults/SWPE	Mean	6.52	4.98	4.8	5.08	4.61
	SD	0.91	0.53	0.42	0.44	0.62
**Predictor Variables**						
ARIA value	Mean	0.22	2.39	4.77	6.87	10.12
	SD	0.28	0.80	0.65	0.56	0.82
Bulk-billing rate	Mean	82.8%	55.7%	52.1%	65.5%	71.4%
	SD	11.2%	13.4%	15.2%	7.0%	20.6%
GP density	Mean	1.21	0.97	0.87	0.77	0.82
	SD	0.33	0.16	0.27	0.17	0.16
Index of disadvantage	Mean	6.80	5.00	4.50	3.61	3.93
	SD	2.31	1.38	0.84	0.22	0.52
% born NESB country	Mean	16.0%	3.9%	4.9%	4.8%	3.9%
	SD	10.4%	1.6%	2.6%	1.5%	1.9%
% female	Mean	50.4%	50.3%	49.5%	48.5%	47.6%
	SD	1.3%	1.0%	1.8%	2.7%	3.1%
Average age (yrs)	Mean	35.22	35.62	33.86	34.09	32.08
	SD	2.45	1.76	2.20	2.32	2.15

**Data Sources**: See Table 1

**Notes**: * Categories based on the following ARIA values: highly accessible = 0 – 1.84, accessible = >1.84 – 3.51, moderately accessible = >3.51 – 5.80, remote = >5,80 – 9.08, very remote = <9.02 – 12. Source: Commonwealth Department of Health and Ageing, Accessibility/Remoteness Index of Australia (ARIA), available at  Accessed 8th January 2000.

### Analysis

Stepwise ordinary least squares (OLS) regression analyses have been undertaken for both models. A forward selection algorithm with an exclusion threshold of p > 0.2 has been used to enter each of the predictor variables. The main predictor variables which were not excluded using this threshold formed the 'stem' and their two-way and three-way interactions were entered sequentially using the same algorithm and exclusion criteria.

## Results

### Decision to consult model

The main predictor variables for the decision to consult model are GP density, bulk-billing rate, socioeconomic disadvantage of the resident population and geographic accessibility (ARIA). Age, gender and NESB status of the resident population are not predictors in this model. The signs of the predictor variables indicate that:

• Higher levels of socioeconomic disadvantage in the resident population are associated with higher levels of the decision to consult index.

• Lower ARIA scores (higher geographic accessibility) are associated with higher levels in the decision to consult index.

• Although the signs for GP density and bulk-billing rates are positive, the three-way interaction term in the model indicates that the relationships between the decision to consult an FFS GP and the supply of GPs and bulk-billing rate are not straightforward. (Table 3)

**Table 3:** Regression model for decision to consult

Model	Unstandardized coefficients		Standardized coefficients	t-value	Significance
	B	Std Error	Beta		

Constant	0.497	0.039	12.897	0.000	
GP density	0.234	0.020	0.559	11.717	0.000
Index of disadvantage	0.018	0.003	0.299	6.458	0.000
Bulk-billing rate	0.127	0.032	0.177	3.977	0.000
ARIA	-0.010	0.006	-0.205	-1.812	0.073
ARIA * GP density * Bulk-billing rate	-0.037	0.008	-0.444	-4.308	0.000

**Notes**: ARIA = population weighted Accessibility/Remoteness Index of Australia

Higher index of disadvantage = higher level of socioeconomic disadvantage

Dependent variable = number of Whole Patient Equivalents (WPEs) per head of population (see Table 1)

Rewriting the regression equation in the following way clarifies the relationship between the decision to consult and the bulk-billing rate.

Decision to consult = 0.497 + 0.234*GP density + 0.018*Index of disadvantage + Bulk-billing rate*(0.127 - 0.037*ARIA*GP density) - 0.010*ARIA

This equation shows that the positive effect associated with any given bulk-billing rate will be greatest in the most highly accessible Divisions (i.e. where ARIA = 0), and the 'turning point' occurs where the bracketed term equals zero (i.e. when. ARIA*GP density = 3.4 and 0.037*ARIA*GP density = 0.127). In the data set used for this analysis, there are twenty-one Divisions where ARIA*GP density is greater than 3.4. The modelling predicts that increasing the bulk-billing rate ceteris paribus in these twenty-one Divisions will lead to a decrease in the number of people consulting an FFS GP within the Division. As can be seen in Table 4, not all these Divisions are characterised by low bulk-billing rates and/or low decision to consult indices.

**Table 4:** Divisions in which an increase in the bulk-billing rate will decrease the number of people consulting an FFS GP

**Geographic Accessibility**	**Bulk-billing rate**	**Index of disadvantage†**	**GP density‡**	**WPEs per head of population¶**
Accessible*				
1. 230	59%	6.8	0.96	0.82
2. 412	73%	4.7	1.19	0.93
3. 413	81%	4.5	1.06	0.85
4. 507	69%	8.0	1.37	0.90
5. 609	47%	5.3	0.89	0.80
Moderately Accessible*				
6. 231	56%	7.2	0.90	0.78
7. 411	51%	5.6	0.96	0.79
8. 509	21%	7.7	1.26	0.84
9. 511	69%	7.1	0.84	0.86
10. 801	62%	5.8	1.07	0.67
Remote*				
11. 241	76%	9.1	0.69	0.76
12. 416	64%	7.3	0.54	0.65
13. 417	64%	7.9	0.73	0.71
14. 512	72%	7.8	0.90	0.74
15. 611	59%	5.3	1.01	0.71
16. 612	58%	7.0	0.78	0.63
Very Remote*				
17. 233	93%	8.9	0.71	0.70
18. 415	57%	6.8	0.65	0.50
19. 610	93%	8.0	1.06	0.38
20. 614	49%	4.8	0.79	0.61
21. 802	65%	6.8	0.89	0.51

**Notes**: * Categories based on the following ARIA values: highly accessible = 0 – 1.84, accessible = >1.84 – 3.51, moderately accessible = >3.51 – 5.80, remote = >5,80 – 9.08, very remote = <9.02 – 12. Source: Commonwealth Department of Health and Ageing, Accessibility/Remoteness Index of Australia (ARIA), available at  Accessed 8th January 2002.

† Higher values indicate higher levels of socioeconomic disadvantage

‡ Vocationally registered and other GPs per 1,000 head of population

¶ WPE = whole patient equivalent (see Table 1)

The standardized beta coefficients in Table 3 indicate that GP density is the most important predictor in the decision to consult model. The regression equation can also be rewritten to emphasise the relationship between utilisation and GP density.

Decision to consult = 0.497 + 0.018*Index of disadvantage + 0.127*Bulk-billing rate + GP density*(0.234 - 0.037*ARIA*Bulk-billing rate) - 0.010*ARIA

The positive effect associated with GP density will be greatest in highly accessible Divisions (ARIA = 0) and the 'turning point' will occur when ARIA*Bulk-billing rate equals 6.3. In the current data set this occurs in the three very remote Divisions: 233, 610 and 802. The modelling predicts that increasing GP density ceteris paribus in these Divisions would lead to a decrease in the number of people consulting an FFS GP – an apparently perverse result.

### Frequency of consultation model

GP density and socioeconomic disadvantage of the resident population do not enter as predictor variables in the frequency of consultation model. As shown in Table 5, the main predictors are the bulk-billing rate squared and the proportion of the resident population born in a non-English speaking background (NESB) country. There are two interaction terms: bulk-billing rate squared*ARIA; and bulk-billing rate squared*Proportion of the resident population born in an NESB country.

**Table 5:** Regression model for the frequency of consultation

Model	Unstandardized coefficients		Standardized coefficients	t-value	Significance
	B	Std Error	Beta		

Constant	4.296	0.120		35.777	0.000
Bulk-billing rate squared	2.767	0.204	0.619	13.539	0.000
Bulk-billing rate squared * ARIA	-0.192	0.025	-0.248	-7.621	0.000
Prop^n ^pop^n ^born NESB country	-4.154	1.841	-0.369	-2.256	0.026
Prop^n ^pop^n ^born NESB country * Bulk-billing rate squared	8.108	2.091	0.675	3.878	0.000

**Notes**: ARIA = population weighted Accessibility/Remoteness Index of Australia

NESB = Non-English Speaking Background

Dependent variable = number of consults per Standardised Whole Patient Equivalent (SWPE – see Table 1)

To emphasise the relationship between the bulk-billing rate and frequency of consultation the regression equation can be written as:

Frequency of consultation = 4.296 + Bulk-billing rate sq*(2.767 + 8.108*Prop^n ^population born NESB country - 0.192*ARIA) - 4.154*Prop^n ^population born NESB country

The positive impact associated with the bulk-billing rate will be highest in the most geographically accessible Divisions (i.e. where ARIA = 0) which have a high proportion of the resident population born in an NESB country. Assuming the proportion of the population born in an NESB country is zero, then the 'turning point' in the relationship between bulk-billing and frequency of consultation will occur when 0.192*ARIA equals 2.767 (i.e. ARIA = 14.4). Since the maximum ARIA value is twelve the 'turning point' will not be reached and the modelling predicts that a ceteris paribus increase in the bulk-billing rate will unambiguously increase the frequency of consultation across all Divisions.

### Performance of the models

**Figure 1:**
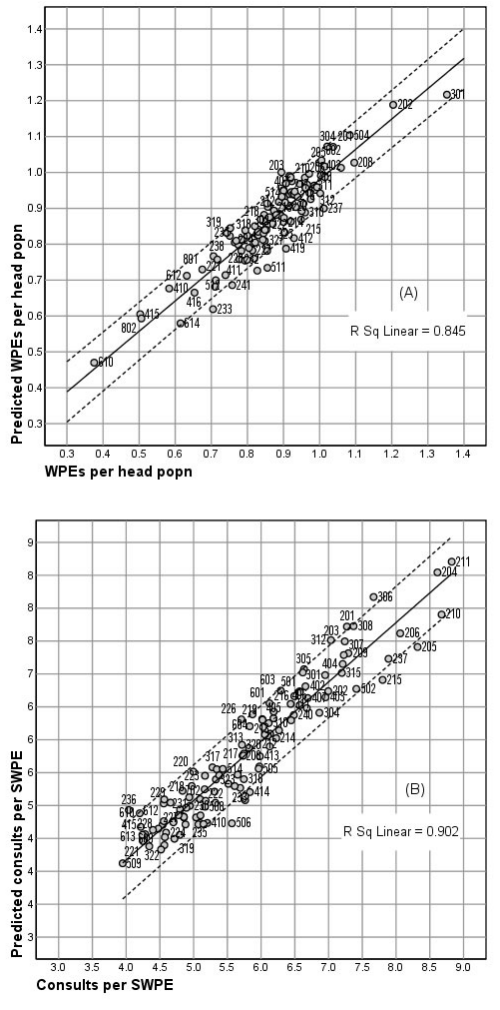
**Performance of the models**. Actual and predicted values of the dependent variables in the regression models (a) Decision to consult, (b) Frequency of consultation.

In both models the residuals are normally distributed and both have good explanatory power. The linear R^2 ^value for the decision to consult is 0.845 and for the frequency of consultation it is 0.902. (Figure 1)

## Discussion

Using publicly accessible data, aggregated to the level of the Division of General Practice, this research explores the associations between the bulk-billing rate, the supply of FFS GPs and utilisation of Medicare-funded GP services in rural and urban areas of Australia. Although the use of aggregated data has been described as a useful 'first-cut method' for getting insights into the 'interplay of macroeconomic variables for the analysis of economic policy', [42] such data has been criticised because it conceals individual differences in economic behaviour [43]. Because the primary aim of this research is to look at the likely impact of the Federal government's *Strengthening Medicare *policy on access to Medicare-funded GP services it is considered an appropriate methodology although there are some caveats that need to be noted.

No inferences can be drawn about the modelled relationships at the intra-Divisional level (i.e. at the individual or postcode level). It also needs to be borne in mind that the data used in the modelling related to a period some six years prior to the introduction of *Strengthening Medicare*. Policy changes in the intervening six years may have changed the nature of the modelled relationships. And a major assumption underlying the analysis is that the allocation of a single patient to different geographic areas is the result of border crossing by the patient. To the extent that the geographic allocation also results from border crossing by GPs (i.e. patients seen by a GP in one Division are linked to provider numbers in a different area) then the extent of border crossing by patients will be overstated. It is not clear from the data to what extent the results are confounded by GPs' border crossing.

Bearing these caveats in mind, the results indicate that the relationship between bulk-billing and utilisation is complex. This complexity arises, in part, because utilisation is a dynamic process involving both the decision to consult a doctor and the frequency of consultation once contact has been established. Each stage in this process involves a unique set of interacting predictor variables. In addition, each Division represents a unique combination of the predictor variables which means that the impact of changes in bulk-billing will differ from Division to Division. Despite this complexity it is possible to draw a number of inferences from the modelling that have important policy implications.

The level of socioeconomic disadvantage in the resident population has no impact on frequency of consultation but higher levels of socioeconomic disadvantage in the resident population are associated with higher decision to consult ratios. These results suggest that, at the Divisional level, the delivery of FFS GP services in the 1998–99 period was responsive to the needs of socioeconomically disadvantaged groups.

In many, but not all, Divisions an increase in the bulk-billing rate will unambiguously increase utilisation of Medicare-funded GP consults within a given period: it will increase both the number of people consulting an FFS GP and the number of visits per patient. However, in a number of Divisions, an increase in the bulk-billing rate will simultaneously increase the number of times existing patients consult and decrease the number of people consulting in a given period. The most straightforward interpretation of this phenomenon is that new patients are 'crowded out' by existing patients' increased use of services.

Increasing the supply of FFS GPs in a Division will, in all but three very remote Divisions, increase the number of people consulting an FFS GP in a given period without impacting on the number of times patients consult. This would mean an increase the total number of FFS GP consults in a Division in a given period and, assuming a stable population, an increase in the number of consults per head of resident population. The positive relationship between supply and the number of people contacting a GP is consistent with both the SID hypothesis and orthodox economic theory, but the modelling also implies that SID is not occurring at the point in the decision-making process where it might be most expected to occur – in the frequency of consultation. These results are consistent with Escarce's findings in relation to surgeons [32]. The reasons for the apparently perverse results in the three very remote Divisions cannot be clarified in this modelling exercise and require a more in-depth analysis to understand the dynamics underlying the result.

## Conclusion

Bulk-billing rates and the supply of FFS GPs are two important supply-side elements in the Australian health care system that are potentially amenable to policy manipulation. To the extent that the *Strengthening Medicare *policy has increased bulk-billing rates, and bearing in mind the qualifications expressed in relation to the data, the modelling indicates that an increase in bulk-billing rates will not necessarily increase access across all Divisions. Unlike the American HIE results which showed no difference in response across geographic areas, in Australia, rurality is clearly important in understanding the relationships between cost, supply and access to medical care and needs to be considered when modelling the impact that policy initiatives on access to FFS GP services.

*Strengthening Medicare *was specifically designed to increase access for financially disadvantaged patients and those less than 16 years of age. The modelling suggests that, in the late 1990s, FFS GPs were responsive to the needs of socioeconomically disadvantaged patients and age was not an important predictor in the utilization of Medicare-funded GP services. If this situation still existed prior to the introduction of the new policy, then it is likely that the impact of the initiative on access to FFS GP services for these two groups of patients would be somewhat restrained.

Finally, geographic differences in bulk-billing rates and the supply of FFS GPs are often taken to be evidence of inequities in the provision of heath services. According to Hancock, social equity requires that the 'provision of health services ... should not discriminate on the grounds of ... rurality or geographical location' [44]. However, policy strivings for equivalence in performance indicators such as bulk-billing rates across geographic areas will not necessarily result in geographic equity of access. If geographic accessibility is not addressed, ensuring equity of access to FFS GP services in Australia would seem to require positive discrimination in favour of rural areas such that the supply of FFS GPs and bulk-billing rates are higher in rural than metropolitan areas.

## Competing interests

This work is funded by an Australian Research Council grant and the North East Victorian Division of General Practice is an industry partner in the research.

## Authors' contributions

SED: participated in the design, implemented the study drafted and revised the manuscript

KA: conceived the study, participated in its design and helped draft and revise the manuscript

DD: participated in the design of the study and revised the manuscript

SP: provided technical advice in the design of the study and revised the manuscript

LG: participated in the design and implementation of the data analysis and revised the manuscript

DV: conceived the study, participated in its design and revised the manuscript

## References

[B1] Pratt A Health Insurance Amendment (100% Medicare Rebate and Other Measures) Bill 2004.

[B2] Department of Health and Ageing (2004). Annual Report 2003–04.

[B3] Senate Select Committee on Medicare Medicare – healthcare or welfare?.

[B4] Senate Select Committee on Medicare Medicare Plus: the future for Medicare?.

[B5] Abbott T Ministerial media releases. ABB178/04. Bulk-billing rates continue to rise.

[B6] Manning WG (1987). Health Insurance and the Demand for Medical Care: Evidence from a Randomized Experiment. American Economic Review.

[B7] Richardson J (1991). The Effects of Consumer Co-payments in Medical Care.

[B8] Richardson J (1987). Does bulk billing cause abuse of Medicare?. Community Health Stud.

[B9] Young AF, Dobson AJ, Byles JE (2001). Determinants of general practitioner use among women in Australia. Soc Sci Med.

[B10] Rosenman SJ, Mackinnon A (1992). General practitioner services under Medicare. Aust N Z J Public Health.

[B11] Knox SA, Britt H (2004). The contribution of demographic and morbidity factors to self-reported visit frequency of patients: a cross-sectional study of general practice patients in Australia. BMC Family Practice.

[B12] Furler JS (2002). The inverse care law revisited: impact of disadvantaged location on accessing longer GP consultation times. Med J Aust.

[B13] Turrell G, Oldenburg BF, Harris E, Jolley DJ, Kimman ML (2003). Socioeconomic disadvantage and use of general practitioners in rural and remote Australia. Med J Aust.

[B14] Richardson J (1981). The Inducement Hypothesis: That Doctors General Demand for Their Own Services. Health, economics, and health economics: proceedings of the World Congress on Health Economics.

[B15] Richardson J, Peacock S Supplier Induced Demand Reconsidered.

[B16] Doessel DP (1998). Is an increased medical workforce a "problem" in the health sector? Theory and evidence. the Nineteenth Australian Conference of Health Economists.

[B17] Dranove D (1988). Demand inducement and the physician/patient relationship. Economic Enquiry.

[B18] Folland S, Goodman AC, Stano M (2001). The Economics of Health and Health Care.

[B19] Rice TH, Labelle RJ (1989). Do Physicians Induce Demand for Medical Service?. Journal of Health Politics, Policy and Law.

[B20] Donaldson C, Gerrard K (1989). Paying general practitioners: shedding light on the review of health services. Journal of the Royal College of General Practitioners.

[B21] Fuchs V (1978). The supply of surgeons and the demand for operations. Journal of Human Resources.

[B22] Fuchs VR, Kramer MJ (1972). Determinants of Expenditures for Physicians' Services in the United Stated, 1948–1968.

[B23] Cromwell J, Mitchell JB (1986). Physician-induced demand for surgery. Journal of Health Economics.

[B24] Phelps CE (1986). Induced Demand – Can We Ever Know Its Extent?. Journal of Health Economics.

[B25] Scott A, Culyer AJ, Newhouse JP (2000). Economics of General Practice. Handbook of Health Economics.

[B26] Grossman M, Culyer AJ, Newhouse JP (2000). The Human Capital Model. Handbook of Health Economics.

[B27] Duan N, Manning W, Morris CN, Newhouse JP (1983). A comparison of alternative models of the demand for medical care. Journal of Business and Economic Statistics.

[B28] Pohlmeier W, Ulrich V (1995). An Econometric Model of the Two-Part Decisionmaking Process in the Demand for Health Care. Journal of Human Resources.

[B29] McGuire TG, Culyer AJ, Newhouse JP (2000). Physician Agency. Handbook of Health Economics.

[B30] Rossiter LF, Wilensky GR (1984). Identification of Physician-Induced Demand. Journal of Human Resources.

[B31] Rossiter LF, Wilensky GR (1983). A Reexamination of the Use of Physician Services: The Role of Physician-Initiated Demand. Inquiry.

[B32] Escarce JJ (1992). Explaining the Association between Surgeon Supply and Utilisation. Inquiry.

[B33] Humphreys JS, Weinand HW (1989). Health status and health care in rural Australia: a case study. Community Health Stud.

[B34] Strong K, Trickett P, Titulaer I, Bhatia K (1998). Health in Rural and Remote Australia.

[B35] Cutler DM, Zeckhauser RJ, Culyer AJ, Newhouse JP (2000). The Anatomy of Health Insurance. Handbook of Health Economics.

[B36] Rice T, Morrison KR (1994). Patient cost sharing for medical services: a review of the literature and the implications for health care reform. Medical Care Review.

[B37] Zweifel P, Manning WM, Culyer AJ, Newhouse JP (2000). Moral Hazard and Consumer Incentives in Health Care. Handbook of Health Economics.

[B38] Review Panel The Future Role of the Divisions Network.

[B39] Australian Government Medicare Benefits Schedule (MBS) Group Statistics Report.

[B40] GISCA ARIA vs RRMA.

[B41] Australian Bureau of Statistics Census of Population and Housing: Socio-economic Indexes for Areas (SEIFA), Australia.

[B42] Stoker TM (1993). Empirical Approaches to the Problem of Aggregation Over Individuals. Journal of Econometric Literature.

[B43] Schwartz S (1994). The Fallacy of the Ecological Fallacy: The Potential Misuse of a Concept and the Consequences. Am J Public Health.

[B44] Hancock L, Hancock L (1999). Rights and markets: What makes sustainable health policy?. Health Policy in the Market State.

